# In-vivo multi-exponential T_2_, magnetization transfer and quantitative histology in a rat model of intramyelinic edema^☆^^☆☆^

**DOI:** 10.1016/j.nicl.2013.06.007

**Published:** 2013-06-22

**Authors:** Kevin D. Harkins, William M. Valentine, Daniel F. Gochberg, Mark D. Does

**Affiliations:** a Institute of Imaging Science, Vanderbilt University, USA; b Department of Pathology, Vanderbilt University, USA; c Department of Radiology and Radiological Sciences, Vanderbilt University, USA; d Department of Physics and Astronomy, Vanderbilt University, USA; e Department of Biomedical Engineering, Vanderbilt University, USA; f Department of Electrical Engineering, Vanderbilt University, USA

**Keywords:** MRI, T_2_, Magnetization transfer, Edema

## Abstract

Two MRI methods, multi-exponential analysis of transverse relaxation (MET_2_) and quantitative magnetization transfer (qMT), were used along with quantitative evaluation of histology in a study of intra-myelinic edema in rat spinal white matter. The results showed a strong linear correlation between a distinct long-T_2_ signal from MET_2_ analysis and the edema water volume fraction as measured by histology, although this analysis overestimated the edema water content by ≈ 100% relative to quantitative histological measurements. This overestimation was reasoned to result from the effects of inter-compartmental water exchange on observed transverse relaxation. Commonly studied MRI markers for myelin, the myelin water fraction (from MET_2_ analysis) and the macromolecular pool size ratio (from qMT analysis) produced results that could not be explained purely by changes in myelin content. The results demonstrate the potential for MET_2_ analysis as well as the limits of putative myelin markers for characterizing white matter abnormalities involving intra-myelinic edema.

## Highlights

• We studied a rat model of intra-myelinic edema induced by hexachlorophene ingestion.
• We used multi-exponential T_2_ (MET_2_) and quantitative magnetization transfer MRI.
• Histology was quantitatively evaluated to measure edema volume and myelin content.
• MET_2_ provides a measure that correlates but overestimates with edema volume fraction.
• MET_2_ measure of edema is affected by microscopic water dynamics.

## 1 Introduction

Many types of white matter lesions appear with high contrast in standard magnetic resonance imaging (MRI) protocols, however, the appearance of lesions is mostly unspecific for diagnosis. Both inflammation and demyelination will lead to an increase in average transverse relaxation time constant (T_2_) (Stanisz et al., 2004; Odrobina et al., 2005), thereby increasing contrast in a T_2_-weighted image. One example where the microstructural basis of T_2_ hyperintensity remains unclear is that seen in pediatric patients on the anti-seizure drug, vigabatrin. In several animal species, vigabatrin has been found to produce intra-myelinic edema (IME) (Cohen et al., 2000), which is characterized by a separation of the lamellae at the intraperiod line of compact myelin. In humans, vigabatrin has been associated with reversible T_2_ hyperintensity in pediatric patients (Dill et al., 2013; Dracopoulos et al., 2010; Pearl et al., 2009), but lack of imaging specificity has left the microanatomical basis for these observations unknown. Dracopoulos et al. (2010) found a decrease in the apparent diffusion coefficient (ADC) of water corresponding to regions of T_2_ hyperintensity, which is consistent with IME (Karaarslan and Arslan, 2008; Righini et al., 2003), but Simao et al. (2011) found a directional dependence to the ADC change which led to the postulate that it resulted from axonal rather than myelinic sources. More recently, it has also been suggested that some observed T_2_ changes could correspond to physiologic rather than potentially pathologic changes (Aguilera-Albesa et al., 2012). From the perspective of MRI method development and evaluation, the unknown cause of T_2_ contrast observed in some patients taking vigabatrin is just one example of the need for establishing a stronger quantitative relationship between tissue microstructure and MRI contrast.

One approach for providing greater specificity to neuronal MRI contrast is through the use of multi-exponential T_2_ (MET_2_) analysis of water proton transverse relaxation, as measured through a multiple spin-echo imaging pulse sequence (Mackay et al., 1994). With this method, the water from within myelin, known as the myelin water fraction (MWF) has been distinguished from water in the intra- and extra-axonal spaces of white matter based on its short T_2_ (Laule et al., 2004; Mackay et al., 1994; Menon et al., 1992; Vasilescu et al., 1978). In addition to the MWF, the full spectrum of transverse relaxation time constants present in a tissue (hereafter, the T_2_ spectrum) provides other information about nervous system microstructure. For example, inflammation in the absence of myelin loss shifts the long-T_2_ components to greater T_2_ values (Stanisz et al., 2004), and an additional long T_2_ signal (> 100 ms) has been observed in the white matter of patients with phenylketonuria (Laule et al., 2007; Sirrs et al., 2007) and in peripheral nerve exhibiting IME (Valentine et al., 2007). In contrast, the loss of myelin may result in a shift of the dominant T_2_ component to longer times, as well as a decrease in the MWF (Does and Snyder, 1996; Odrobina et al., 2005; Stewart et al., 1993). Recent work in spinal white matter and nerve has also shown T_2_-spectral sensitivity to microstructural dimensions, such as axon diameter and myelin thickness, through their effect on inter-compartmental water exchange (Dortch et al., in press; Dula et al., 2010; Harkins et al., 2012; Levesque and Pike, 2009; Sled et al., 2004). Thus, MET_2_ offers a promising approach to characterizing nervous system abnormalities with greater microstructural specificity.

Another approach to providing micro-structural specificity to white matter MRI is through quantitative magnetization transfer (qMT) imaging (Gochberg and Gore, 2007; Sled and Pike, 2001; Yarnykh, 2002). This approach attempts to estimate the relative concentration of macromolecular protons through the exchange of magnetization between these protons and water. Because of the relatively high concentration of phospholipids and proteins in myelin compared to both intra- and extra-axonal spaces, an observed reduction of macromolecular pool size by qMT is reasoned to reflect a loss of myelin (Kucharczyk et al., 1994; Ou et al., 2009; Stanisz et al., 2001; Tozer et al., 2005). However, qMT may be less informative about microstructure than MET_2_ when myelin content does not change (Dula et al., 2010; Levesque and Pike, 2009).

Given recent developments that make robust measurement of the MET_2_ (Guo et al., in press; Lebel and Wilman, 2010; Prasloski et al., 2012a,b) relatively rapid and suitable for multi-slice or 3D acquisitions, and established clinically practical qMT methods (Sled and Pike, 2001; Smith et al., 2006; Yarnykh, 2002), further experimental studies that quantitatively evaluate the white matter micro-structural changes that underlie T_2_ and qMT contrast changes are warranted. This work reports evaluations of the specificity of MET_2_ and qMT to IME induced by hexachlorophene (HCP) intoxication in rat spinal cord.

## 2 Materials and methods

### 2.1 Animals

Animal studies were completed in compliance with the Vanderbilt University Institutional Animal Care and Use Committee. Twenty-four female Sprague Dawley rats (pre-study weight 208–255 g) were split into three groups of 8 rats each and fed a diet containing various amounts of HCP, which is a toxin known to cause IME lesions histologically similar to those caused by vigabatrin (Graham, 1989). One group (referred to as HCP600) consumed a diet with 600 ppm HCP added to normal chow (TestDiet/LabDiet, Richmond, IN), another group (HCP300) consumed 300 ppm HCP, and a third group (CONTROL) consumed the same chow without added HCP. Doses were chosen to deliver approximately the same toxicity as 20 mg/kg/day and 40 mg/kg/day of HCP via oral gavage (Kennedy and Gordon, 1976), and similar to previous rat studies of HCP intoxication (Cammer et al., 1975; Kimbrough and Gaines, 1971; Lampert et al., 1973). All rats were held to the modified diet for four days prior to MR imaging, and the neurological status and weight of each rat were monitored daily. For all MRI studies, rats were anesthetized with isoflurane, respiration rate was continuously monitored, and a computer controlled external heater was used to maintain body temperature near 37 °C.

### 2.2 Magnetic resonance imaging

MR imaging was performed on a 9.4 T, 31-cm horizontal bore magnet with Varian/Agilent DirectDrive console (Santa Clara, CA) using a 38-mm Litz quadrature coil (Doty Scientific, Columbia SC) for radiofrequency excitation and signal reception. From a sagittal scout image, a 1.5-mm thick slice was selected transverse to the long axis of the cervical spinal cord at the C2 level for further evaluation. Multi-exponential T_2_ measurements were made using an inversion-recovery prepared multiple spin-echo imaging sequence as previously described (Harkins et al., 2012), with TR = 6 s. An initial 32 echoes were collected with a first echo time of 7.4 ms and an echo spacing of 9 ms, and final 8 echoes were collected with an echo spacing of 50 ms. Images were encoded with a 128 × 128 sampling over a 25.6 × 25.6 mm^2^ field-of-view. The inversion-recovery preparation (inversion delay, TI = 2 s) was used to null signal from the cerebrospinal fluid, which otherwise caused significant motion-induced ghosting in long echo-time images. Signals from eight excitations were averaged resulting in an acquisition time ≈ 100 min. Quantitative magnetization transfer measurements were made with the same imaging geometry using a selective inversion-recovery prepared fast spin echo sequence (Gochberg and Gore, 2007) with a 1.5-ms hard inversion pulse, an echo train length of 16 and echo spacing of 5.6 ms. Images were acquired at 25 inversion times pseudo-logarithmically spaced between 3.5 ms and 10 s. The repetition pre-delay was fixed at 3.5 s and two signals were averaged resulting in an acquisition time ≈ 25 min. For both sequences, after 2 × zero-padded reconstructions, the nominal in-plane resolutions were 100 μm in each direction.

**Fig. 1:**
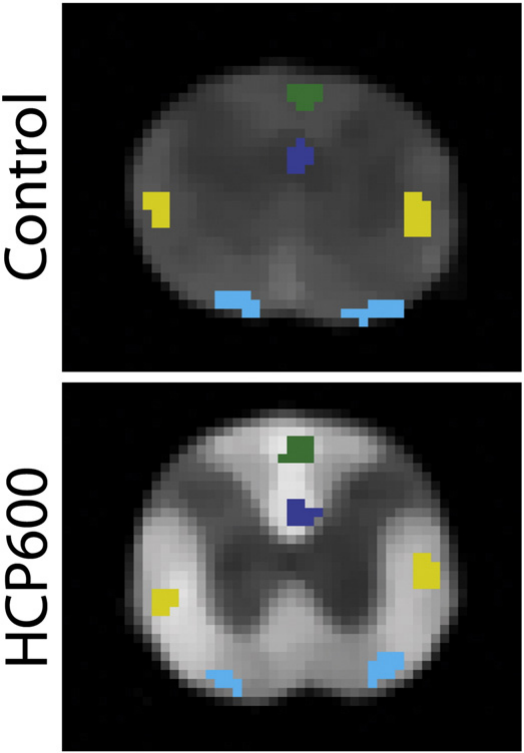
Multi-exponential T_2_ images from control and HCP600 rats at TE = 88.4 ms, showing hyperintensity in white matter regions of HCP intoxicated animals compared to controls. Example ROIs are shown for the dorsal cortical spinal tract (dCST, blue), the funicilus gracilis (FG, green), the rubrospinal tract (RST, yellow), and the vestibulospinal tract (VST, teal). Note that the relatively low white/gray contrast in the control spinal cord, which may be counterintuitive for a T_2_-weighted image, is consistent with previous characterization of rat spinal cord (Carvlin et al., 1989; Narayana et al., 1999). Images were scaled to similar gray matter intensity.

All data analysis was performed using MATLAB (Mathworks, Natick, MA). Images were cropped to view the spinal cord, and regions of interest (ROIs) were drawn for each of four spinal white matter tracts: the dorsal cortical spinal tract (dCST), the funicilus gracilis (FG), the rubrospinal tract (RST) and the vestibulospinal tract (VST) (all as shown in Fig. 1) on the shortest echo time image. The mean signal magnitudes from each ROI in the MET_2_ images were fitted to the sum of up to three decaying exponential functions, each with a Gaussian spectrum of log-spaced time constants, and a non-negative amplitude constraint (Stanisz and Henkelman, 1998). For each animal and ROI, the number of Gaussian-shaped T_2_ components was determined by F-tests comparing χ^2^ residuals between fits with 1, 2, or 3 components (Bevington and Robinson, 2003). Across all animals and ROIs, a k-means algorithm clustered the T_2_ component values into short, intermediate, and long. For each animal and tract, the fraction of signal with short T_2_ was defined as the MWF, consistent with previous definitions of this term (Mackay et al., 1994), and the fraction of the signal with long T_2_ was defined analogously as the edema water fraction (EWF). Intra-ROI standard error in the EWF and MWF was estimated by voxel-by-voxel analysis of the MET_2_ signal.

Since qMT images were acquired sequentially, images at all inversion-times were first rigidly co-registered (Viola and Wells, 1997). Because the qMT images were not exactly co-registered to the spin echo images, ROIs for qMT analysis were manually re-drawn to match the ROIs used in the MET_2_ analysis. The mean signal magnitudes from each ROI were then fitted as a function of inversion time to a 5-parameter model describing longitudinal relaxation in a coupled two-pool model (Li et al., 2010). Three of these fitted parameters, T_1_, the ratio of macromolecular pool size to free water pool size, defined here as the pool-size ratio (PSR), and an exchange rate (k_mf_) were tabulated across all ROIs and rats. As above, intra-ROI standard error was estimated by voxel-by-voxel analysis of the qMT signal.

### 2.3 Histology and light microscopy

After MR imaging, three animals from each group were further processed for histological analysis. Animals were sacrificed by perfusion fixation—an initial flush of phosphate-buffered saline (PBS) was pushed directly into the left ventricle, followed by ≈ 15 min perfusion of a 4% glutaraldehyde and 0.5% paraformaldehyde in PBS. The cervical spinal cord was dissected, cut to ≈ 1 cm long segment surrounding the imaging slice and immersed in the same fixative solution for at least 48 h before being moved into PBS. Samples were post-fixed in 1% osmium tetroxide in cacodylate buffer, dehydrated in graded ethanol, and embedded in an epoxy resin. From each sample, a 1-μm thick section was cut axially from the middle of the embedded spinal cord sample (the approximate location of MRI measures) and stained with toluidine blue for evaluation by light microscopy. The thick sections of spinal cord were evaluated by light microscopy on an Olympus BX41 microscope equipped with an Optronics Microfire digital camera. A minimum of two histological images was collected from each of the aforementioned white matter tracts (dCST, FG, RST and VST) and digitized into a 1600 × 1200 grid over a 120 × 90 μm^2^ field of view.

Histological images were digitally smoothed with an edge-preserving anisotropic diffusion filter, enhanced with adaptive histogram equalization, and semi-automatically segmented using a region growing routine into regions of myelin (my), edema (ed), and intra/extra-axonal space (ie). For each tract, the segmented histology was used to tabulate the volume fraction, *V*, of each compartment. Then, with literature values for relative water density within each compartment (van der Knaap et al., 2005), (ρ_my_ = 0.4, ρ_ie_ = 0.8, ρ_ed_ = 1.0), the estimated water content fraction was defined as

(1) $$W=V_{\text{my}}\rho_{\text{my}}+V_{\text{ie}}\rho_{\text{ie}}+V_{\text{ed}}\rho_{\text{ed}}\text{,}$$

and for each compartment was defined as

(2) $$W_{x}=\frac{V_{x}\rho_{x}}{W}$$

where subscript “*x*” represents either myelin, intra/extra-axonal space, or edema. In addition, the number and area of individual lesions were tabulated, which provided estimates of mean lesion density and mean lesion diameter (computed from area based on a circular geometry).

## 3 Results

**Fig. 2:**
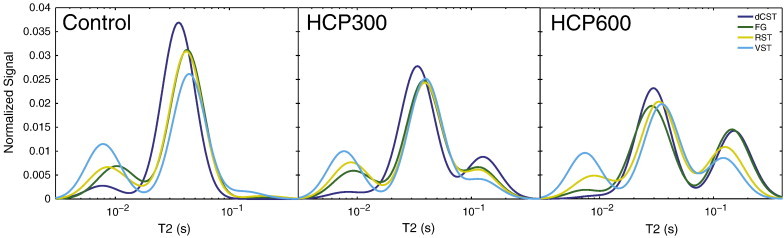
Normalized T_2_ spectra averaged across animals in the three groups—control, HCP300 and HCP600. A third T_2_ component is a result of HCP intoxication, and the signal fraction increases with the level of intoxication.

**Fig. 3:**
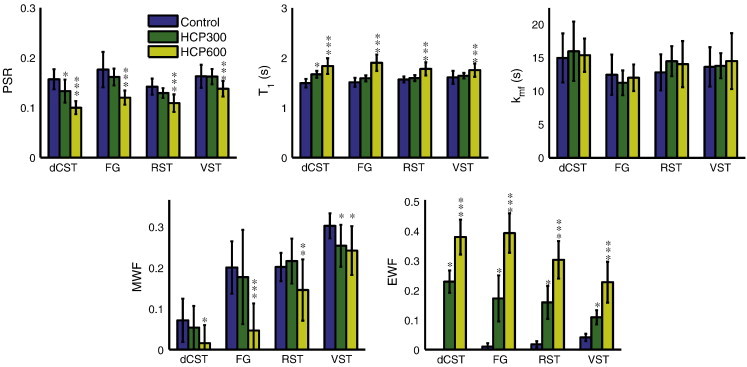
MR imaging metrics: pool size ratio (PSR), T_1_ relaxation time-constant, macromolecular exchange rate (k_mf_), myelin water fraction (MWF), and edema water fraction (EWF) in four spinal cord tracts of the spinal cord in all eight rats from each of the control, HCP300 and HCP600 groups. Error bars indicate inter-animal standard deviation. Asterisks indicate: * HCP300 or HCP600 is statistically significant (P < 0.05) compared to control, ** HCP600 is statistically significant (P < 0.05) compared to HCP300, and *** HCP600 is statistically significant (P < 0.05) compared to control and HCP300.

Representative T_2_-weighted spin-echo images (echo time = 88.4 ms) are shown in Fig. 1 for both control and HCP600 rats, where images have been median-filtered and scaled to similar gray matter brightness. The white matter hyper-intensity in the images from the HCP600 rats relative to control rats suggests the presence of longer-lived T_2_ signals, and these signals are apparent in the T_2_ spectra from the two HCP groups, as shown in Fig. 2. Normal white matter is typically characterized by two T_2_ components (Stewart et al., 1993), as found in the control animals, but the HCP-fed rats exhibited a third T_2_ component (T_2_ > 100 ms), defined here as the edema water component, with the signal fraction that increased with the severity of HCP intoxication. This increased EWF with HCP intoxication is presented in Fig. 3, which shows mean ± inter-animal standard deviation (SD) values of PSR, T_1_, k_mf_, MWF, and EWF for each tract and diet group. Small but statistically significant decreases in PSR and MWF and increases in T_1_ were also observed between control and HCP intoxicated rats. There were no statistical differences in k_mf_ between groups.

**Fig. 4:**
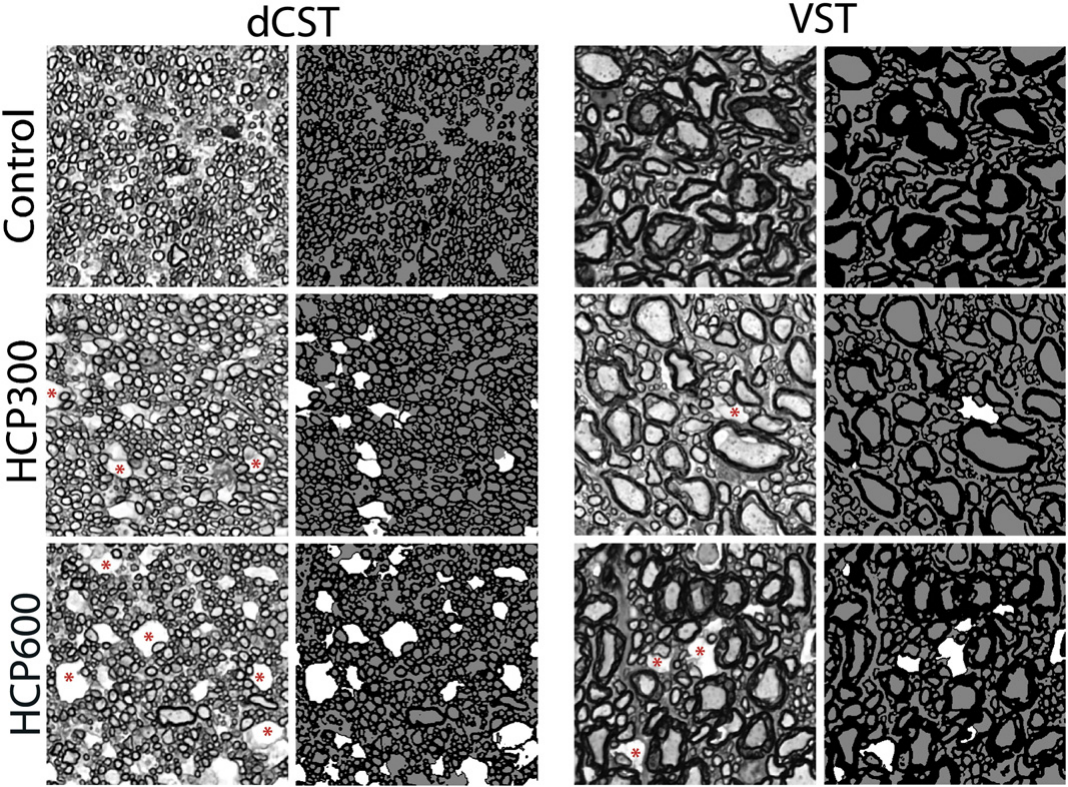
Example histology from dCST and VST spinal cord tracts of control and HCP fed animals. FOV = 30 × 30 μm^2^. Red asterisks indicate a few regions of intramyelinic edema. The histology was segmented into regions of myelin (black), intra/extra-axonal space (gray), and intramyelinic edema (white).

**Fig. 5:**
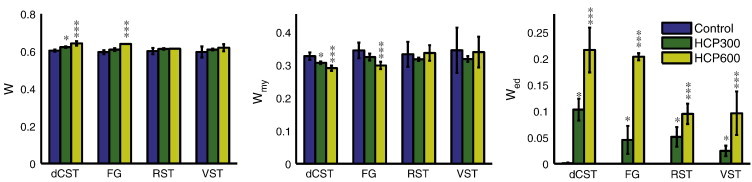
Histology based water content (*W*), myelin water content fraction (*W*_my_), and edema water content fraction (*W*_ed_) in four spinal cord tracts of the spinal cord in three rats from each of the control, HCP300 and HCP600 groups. Error bars indicate inter-animal standard deviation determined from voxel-by-voxel analysis. Asterisks indicate: * HCP300 is statistically significant (P < 0.05) compared to control, *** HCP600 is statistically significant (P < 0.05) compared to control and HCP300.

Example histology and segmented histology from the dCST and VST tracts in control and HCP fed rats are shown in Fig. 4. The cross-sectional area of analyzed histology for each rat and tract represents ≈ 12% of the mean ROI cross-sectional area analyzed with MRI. Red asterisks identify some occurrences of IME, characteristic of HCP intoxication. Across all tracts in both HCP300 and HCP600 animals, IME lesions exhibited a circular shape with diameter mean ± SD = 3.1 ± 0.4 μm. There was no statistical difference (P < 0.05) in average lesion diameter between tracts or HCP dose; however, the average lesion density (mean ± SD number of lesions per square mm) present was greater in HCP600 (1651 ± 201, 1291 ± 286, 545 ± 162, and 497 ± 150 in the dCST, FG, RST and VST respectively) compared to HCP300 (776 ± 97, 469 ± 345, 344 ± 178, and 142 ± 50 in the same tracts) in every tract (P < 0.05). The mean ± inter-animal SD of histology-derived water content fraction (*W*) as well as compartment specific myelin and edema water content fractions (*W*_my_ and *W*_ed_, respectively) are shown in Fig. 5. In accord with the MRI observations of EWF, MWF and PSR, the histology showed large increases in *W*_ed_ in all tracts of HCP300 and HCP600 animals, and relatively small or insignificant changes in *W* and *W*_my_.

**Fig. 6:**
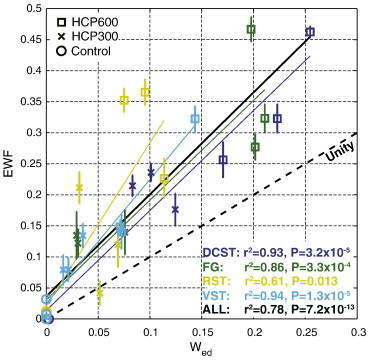
Scatter plot comparing measures of edema water fraction from MET_2_ imaging (EWF) with histology (*W*_ed_) within four spinal cord tracts. Individual data points are plotted for animals for which both MRI and quantitative histology were obtained (3 per treatment group). Error bars indicate intra-ROI standard error, as estimated through voxel-by-voxel fitting. Solid lines draw linear regressions for individual tracts (colored to match the tract data points) as well as all tracks considered together (black, r^2^ = 0.78).

**Fig. 7:**
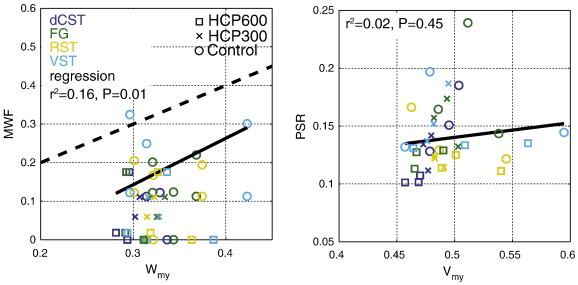
Scatter plots comparing measures of myelin content from MRI and histology. On the left are plots of myelin water fraction from MET_2_ imaging (MWF) vs histological estimates of myelin water volume fraction (*W*_my_). On the right are plots of macromolecular pool size from qMT imaging (PSR) with histological measures of myelin volume fraction (*V*_my_). Individual data points are plotted for animals for which both MRI and quantitative histology were obtained (3 per treatment group). Solid lines show best fit linear regressions for all data, which revealed a weak but significant correlation between MWF and *W*_my_ (r^2^ = 0.16, P < 0.05) and no significant correlation between PSR and *V*_my_ (r^2^ = 0.02, P > 0.05). The average intra-ROI standard error was small compared to the marker size, with average values of 0.016 for the MWF and 0.0051 for the PSR.

The relationships between MRI metrics and histology are further shown in Figs. 6 & 7. Individual points are plotted for each tract and rat from which both MRI and histology metrics were available (± intra-ROI standard error, as determined by voxel-by-voxel analysis). The lines show the best-fit linear relationship between MRI-derived and histology-derived metrics for data from individual tracts (colored according to the tract) and for all data (black). Fig. 6 shows significant correlations between EWF and *W*_ed_, with a large overall coefficient of determination (r^2^ = 0.78). However, this figure also shows that EWF overestimates *W*_ed_ by approximately a factor of 2 × on average. In contrast, Fig. 7 shows only a weak correlation between MWF and *W*_my_ and no significant correlation between PSR and *V*_my_.

## 4 Discussion

In this study, HCP was introduced into rat diets in order to alter the microstructure of white matter within the central nervous system. Parameters from MET_2_ and qMT measurements were compared with quantitative evaluations of histology within several tracts within the spinal cord of normal and intoxicated rats to investigate the specificity of these MRI metrics to variations in white matter microstructure. Compared with relatively unspecific contrast change in a T_2_-weighted image, the use of MET_2_ analysis demonstrated that the increased T_2_ contrast was due to the addition of long T_2_ signal, rather than the loss of short T_2_ signal. Further, the significant correlation between the MRI- and histology-derived measures of edema, as shown in Fig. 6, demonstrates the utility of MET_2_ measurements for quantitative evaluation of the severity of IME. These findings are in accord with and extend findings of several previous studies of water proton relaxation changes in experimental models of IME.

Spectroscopic experiments of excised rat white matter following triethyltin intoxication (Go and Edzes, 1975), known to cause IME, demonstrated correlations between mono-exponential measures of T_1_ and T_2_ with bulk water content, and observed the addition of a long T_2_ component in white matter regions with IME lesions. Using similar methods, Naruse et al. also observed a bi-exponential T_2_ in white matter with triethyltin-induced IME, and found a close correspondence between the time courses of the long T_2_ value and the water content following intoxication (Naruse et al., 1982). Also, in peripheral nerve, IME resulting from dithiocarbamate intoxication has been associated with increased long-T_2_ signals in spectroscopic studies (Valentine et al., 2007). Various preclinical imaging studies have also demonstrated the relationship between relaxation time constants and IME (Barnes et al., 1986, 1987; Jackson et al., 1994; Kinoshita et al., 2000; Lorenzo et al., 1989), and at least one study showed both an increased T_2_ and signal fraction of long-T_2_ signal from a bi-exponential T_2_ analysis (Barnes et al., 1987).

Thus, there is a long-standing appreciation that IME will substantially alter water proton relaxation and generate the addition of a distinct long T_2_ signal component, but the correlation of the size of this signal component to the volume fraction of edema has not previously been established. Fig. 6 demonstrates a significant correlation between the EWF, as measured by MET_2_ MRI, and *W*_ed_, the histological measure of edema water fraction, but it also shows that the EWF is consistently greater than *W*_ed_. This mismatch cannot be simply explained by differential T_1_ weighting on each T_2_ signal component, because the edema water must have a longer T_1_ than the rest of the tissue (as evidenced by the observed increases in bulk T_1_ in HCP rats), so T_1_-weighting effects would result in a reduced, not increased estimate of EWF.

Alternatively, *W*_ed_ may have been systematically underestimated due to limitations of histological analysis. With any type of histological analysis, there is the risk of sampling bias due to the relatively small amount of tissue that can be examined, especially for cases where tissue changes appear in a few focal locations. For the case of HCP intoxication, lesions are known to be distributed diffusely within the spinal cord (Graham, 1989), so their density is not expected to vary substantially over the 1.5 mm thick MRI slice. Also, a relatively large areal fraction of the slice was analyzed by histology (≈ 12%) so it is unlikely that particularly large lesions were systematically missed by histology across multiple animals. In addition to sampling error, the process of preparing tissue for histological analysis may alter compartment volumes. Extracellular volume can decrease during perfusion fixation, although this effect may also be reversed by post-fixing with osmium tetroxide (Van Harreveld and Khattab, 1968). Further, hard plastic embedding is known to cause a 2–4% areal shrinkage of tissue (Oorschot et al., 1991; Tang and Nyengaard, 1997), which may manifest to a greater extent in regions of edema. It seems unlikely that these effects account for the factor of two differences between MRI and histological estimates of edema water, but the ultimate accuracy of the histological measures of compartment sizes is unknown.

Aside from systematic measurement errors, another factor that might explain the larger values of EWF compared with *W*_ed_ is the effect of inter-compartmental water exchange on the observed T_2_ spectrum. The mathematical underpinning of exchange between two relaxing components is well established—as the rate of water exchange increases between two compartments, the amplitude of the faster-relaxing signal components diminishes, while the amplitude of the slower-relaxing component increases (Woessner, 1996; Zimmerman and Brittin, 1957). Thus, in the present case, water exchange between the vacuoles comprising the IME and the surrounding tissue will result in a long T_2_ component with amplitude larger than the water volume fraction of all the vacuoles. The size of vacuoles observed in this study (≈ 3 μm diameter) was small compared to the expected diffusion distance of free water over the transverse relaxation time of edema water, so it is reasonable to expect some effect of water exchange, but can this effect account for the EWF ≈ 2 × *W*_ed_?

For simplicity, assume water exchanges only between the vacuoles and the intra/extra-axonal compartment space. Then consider a two-pool system with intrinsic T_2_s = 50 ms and 150 ms, and 75% of the signal in the short T_2_ pool, which roughly mimics the non-myelin water compartments observed in the HCP rats. With these numbers and the aforementioned mathematical descriptions of relaxation in two exchanging pools, it is easy to calculate that with a residence time constant of the long-T_2_ pool only as short as 200 ms, the observed transverse relaxation will be bi-exponential with two equally sized components. That is, the observed long T_2_ component amplitude will be 2 × the size of the long-T_2_ water pool volume fraction. Given the relatively small size of the vacuoles that comprise IME, a 200 ms residence time constant is not especially short, so these rough calculations indicate that water exchange is a plausible explanation for the two fold overestimation of *W*_ed_ by EWF.

While EWF proved a sensitive and relatively specific measure of IME, neither of the two more commonly studied probes of myelin – MWF or PSR – provided much insight into the micro-structural or -compositional changes resulting from HCP intoxication. Both MWF and PSR changed significantly due to HCP intoxication (Fig. 3), and these changes, if looked at in isolation, might be interpreted as due to decreases in myelin content. However, Fig. 5 demonstrates that there was little or no significant change in the myelin volume fraction due to HCP intoxication, nor was there an overall increase in water content which would result in reduced MWF and PSR by dilution. Unlike the case of interstitial edema, where water content increases and myelin volume fraction is displaced, the IME lesion formed at the expense of intra- and extra-axonal space, resulting in little or no changes in total water content change or myelin volume fraction.

The difference in the presentation of interstitial and intramyelinic edema is also illustrated in the studies of multiple sclerosis and phenylketonuria (Laule et al., 2007; Sirrs et al., 2007)—multiple sclerosis is known to cause interstitial edema, and is present in MET_2_ analysis as a long T_2_ component that is thought to vary in amplitude with the volume of interstitial water. In contrast, phenylketonuria, which is thought to result in IME lesions (Anderson and Leuzzi, 2010) produced consistent long T_2_ signal fractions (Laule et al., 2007) and small but significant changes in water content (Sirrs et al., 2007).

A scatter plot of MWF vs *W*_my_ (Fig. 7, left) reveals a weak (r^2^ = 0.16) but significant (P < 0.05) linear correlation, if all tracts and rats are considered. A previous study found much stronger correlation between MWF and histological measures of myelin content (Laule et al., 2006), which agreed with numerous prior qualitative evaluations (Does and Snyder, 1996; Moore et al., 2000; Odrobina et al., 2005; Stewart et al., 1993). However, all of those studies included comparison of normal tissues with demyelinated and/or non-myelinated tissues. The weaker correlation found here is likely due to the small range in myelin content within the spinal cord tracts analyzed, and consistent with previous studies of MWF in normal rat spinal cord (Dula et al., 2010; Harkins et al., 2012) which showed wide variations in MWF across white matter tracts with similar myelin volume fractions. The MWF variation was postulated to result from variations in water exchange rate between myelin and non-myelin spaces. Similarly, in the present case, the formation of IME may result in more exchange of myelin water with long-T_2_ water, which would then diminish the MWF. This effect would be greater in tracts with thinner myelin (dCST and FG), which agrees with the MWF observations shown in Fig. 3.

In contrast to MWF, no significant correlation was found between PSR and *V*_my_ (r^2^ = 0.02, P > 0.4; Fig. 7, right). This observation is not surprising, given the relatively narrow range of myelin content changes resulting from HCP intoxication, and the fact that PSR is relatively insensitive to variations in the rate of water exchange between tissue compartments (Dula et al., 2010; Levesque and Pike, 2009). The cause of the small but significant decrease in PSR with HCP intoxication is not clear, but may reflect limits of a simple two-pool model of tissue (free water and macromolecules) to report on macromolecular content change when tissue water becomes heterogenous in T_1_, as may be the case in with development of IME. Also, as postulated previously (Stanisz et al., 2004), PSR changes may reflect tissue pH changes, which has been shown to affect qMT measures in model myelin systems (Kucharczyk et al., 1994).

## 5 Conclusion

White matter lesions characterized by IME exhibit a long-T_2_ signal component with amplitude (the EWF) that correlates with the severity of the IME, as measured histologically by the edema water volume fraction. However, much like MWF, EWF appears to be strongly affected by inter-compartmental water exchange, so care must be taken when interpreting results. Because the IME lesions in this study did not result in a substantial loss of myelin, previously established MRI reporters of myelin content, MWF (by MET_2_) and PSR (by qMT) were not fully explained by changes in myelin content. Therefore, for characterizing white matter abnormalities where it is not known a priori that myelin loss is the primary lesion, such as those observed in pediatric patients taking vigabatrin, full T_2_-spectral analysis, which provides measures of both myelin and edema contents, may be the most informative single protocol.
